# Safety and efficacy of chronic weekly rozanolixizumab in generalized myasthenia gravis: the randomized open-label extension MG0004 study

**DOI:** 10.1007/s00415-025-12958-9

**Published:** 2025-03-19

**Authors:** Vera Bril, Artur Drużdż, Julian Grosskreutz, Ali A. Habib, Henry J. Kaminski, Renato Mantegazza, Sabrina Sacconi, Kimiaki Utsugisawa, Tuan Vu, Marion Boehnlein, Maryam Gayfieva, Bernhard Greve, Franz Woltering, John Vissing, on behalf of the MG0004 study investigators, Rodrigo Álvarez-Velasco, Radwa Aly, Henning Andersen, Giovanni Antonini, Aramide Balogun, Ruggero Barnabei, Said Beydoun, Franz Blaes, Silvia Bonarino, Anna Boss Soevang, Nazibrola Botchorishvili, Stephan A. Botez, Ivo Bozovic, Paulina Budzinska, Pietro Businaro, Lucia Campetella, Ana Belen Cánovas, Carlos Casasnovas, Hou-Chang Chiu, His-Chieh Chou, Adam Comer, Elena Cortés Vicente, Roberto D’Angelo, Lubna Daniyal, Annie Dionne, Péter Diószeghy, Laura Fionda, Denis Flemm, Rita Frangiamore, Manuela Gambella, Rachana K. Gandhi Mehta, Matteo Garibaldi, Matteo Gastaldi, Christian Geis, Hannah George, Stefan Gingele, Monica Grau Martin, Yuh-Cherng Guo, Gerardo Gutiérrez Gutiérrez, Francesco Habetswallner, Lina Hassoun, Sonja Holm-Yildiz, Faraz Hussain, Francisca Iniesta, Viktoriya Irodenko, Marina Janelidze, Min Kang, Chafic Karam, Denis Korobko, Sergey Kotov, Michal Kretkowski, Nana Kvirkvelia, Antonio Lauletta, Yi-Chung Lee, Luca Leonardi, Kore Liow, Arnau Llauradó Gayete, Sara Llufriu, Catherine Lomen-Hoerth, Jan D. Lünemann, Lorenzo Maggi, Eugenia Martínez Hernández, Gianvito Masi, Marion Masingue, Rami Massie, Marco Masullo, Federico Mazzacane, Nora Möhn, Stefania Morino, Kelsey Moulton, Tahseen Mozaffar, Elene Nebadze, Velina Nedkova-Hristova, Eduardo Ng, Ekaterina Novikova, Izabella Obál, Anita Palsgård, Claudia Papi, Lorena Pérez, Stojan Peric, Mikhail Petrov, Nicolai Rasmus Preisler, Giorgia Querin, Konrad Rejdak, Kourosh Rezania, Elena Rinaldi, Rita Rinaldi, Michael H. Rivner, Annekathrin Roediger, Laura Rosow, Simone Rossi, Elena Rossini, Stephen Ryan, Lotte Sahin Levison, Albert Saiz, Maria Salvado, Daniel Sánchez-Tejerina, Margret Schwarz, María Sepúlveda, Khema R. Sharma, Sheetal Shroff, Olga Sidorova, Guilhem Solé, Javier Sotoca, Mads Stemmerik, Aleksandar Stojanov, Tanya Stojkovic, Kai Su, Sebastian Szklener, Alexander Tsiskaridze, Laura Tufano, Michaela Tyblova, Eiko Uenaka, Astrid Unterlauft, Gabriel Valero, Fiammetta Vanoli, Tamar Vashadze, Nuria Vidal Fernández, Marie-Hélène Violleau, Nicolas Weiss, Nanna Witting, Jiann-Horng Yeh, Leila Zaidi, Leonid Zaslavskiy, Jana Zschüntzsch

**Affiliations:** 1 Ellen and Martin Prosserman Centre for Neuromuscular Diseases, Toronto General Hospital, University of Toronto, Toronto, Ontario Canada; 2 Department of Neurology, Municipal Hospital, Poznań, Poland; 3 Precision Neurology of Neuromuscular Diseases, Department of Neurology, University of Lübeck, Lübeck, Germany; 4 MDA ALS and Neuromuscular Center, Department of Neurology, University of California, Irvine, Orange, CA USA; 5 Department of Neurology and Rehabilitation Medicine, George Washington University, Washington, DC USA; 6 Department of Neuroimmunology and Neuromuscular Diseases, Fondazione IRCCS, Istituto Nazionale Neurologico Carlo Besta, Milan, Italy; 7 Université Côte d’Azur, Peripheral Nervous System and Muscle Department, Pasteur 2 Hospital, Centre Hospitalier Universitaire de Nice, Nice, France; 8 Department of Neurology, Hanamaki General Hospital, Hanamaki, Japan; 9 Department of Neurology, University of South Florida Morsani College of Medicine, Tampa, FL USA; 10 UCB, Monheim, Germany; 11 UCB, Slough, UK; 12 Copenhagen Neuromuscular Center, Department of Neurology, Rigshospitalet, University of Copenhagen, Copenhagen, Denmark

**Keywords:** Myasthenia gravis, Rozanolixizumab, FcRn inhibitor, Phase 3 clinical trial

## Abstract

**Background:**

In the Phase 3 MycarinG study (NCT03971422), six once-weekly subcutaneous infusions of rozanolixizumab significantly improved myasthenia gravis (MG)-specific outcomes versus placebo in patients with acetylcholine receptor or muscle-specific tyrosine kinase autoantibody-positive generalized MG (gMG). Following completion of MycarinG, patients could enroll in the open-label extension MG0004 study (NCT04124965) to receive chronic weekly rozanolixizumab.

**Methods:**

Patients were re-randomized 1:1 to once-weekly rozanolixizumab 7 or 10 mg/kg for up to 52 infusions. The primary endpoints were the occurrence of treatment-emergent adverse events (TEAEs) and TEAEs leading to rozanolixizumab discontinuation. After ≥6 visits/infusions patients could switch to the MG0007 study (NCT04650854) to receive cyclic rozanolixizumab treatment.

**Results:**

In MG0004, 70 patients received rozanolixizumab 7 mg/kg (*n* = 35) or 10 mg/kg (*n* = 35). Mean treatment duration was 22.9 and 23.7 weeks, respectively, due to rollover into MG0007. TEAEs were reported in 60/70 (85.7%) patients; most were mild/moderate. The most frequently reported TEAEs were headache (25/70 [35.7%]), diarrhea (13/70 [18.6%]) and decreased blood immunoglobulin G (11/70 [15.7%]). There were no opportunistic, serious or severe infections, serious or severe hypersensitivity or injection-site reactions, any anaphylactic reactions or albumin or lipid abnormalities. Maximum mean reduction from baseline in MG Activities of Daily Living score was 3.1 in the 7 mg/kg group and 4.1 in the 10 mg/kg group.

**Conclusion:**

Chronic weekly rozanolixizumab for up to 52 infusions was generally well tolerated, and clinically relevant improvements across MG-specific outcomes were maintained, supporting the long-term use of rozanolixizumab in patients with gMG.

**Trial registration:**

NCT04124965 (registered October 11, 2019).

**Supplementary Information:**

The online version contains supplementary material available at 10.1007/s00415-025-12958-9.

## Introduction

Generalized myasthenia gravis (gMG) is a rare, chronic, neuromuscular disorder characterized by fluctuating muscle weakness and fatigue, including infrequent but potentially life-threatening myasthenic crises [1, 2]. Muscle weakness can vary between individual muscles and muscle groups and worsens with repetitive muscle movement; the unpredictable nature of symptoms also results in a considerable disease burden for patients [2–5]. gMG is caused by pathogenic autoantibodies that bind to functionally important proteins at the postsynaptic membrane of the neuromuscular junction, including the acetylcholine receptor (AChR) and muscle-specific tyrosine kinase (MuSK). Approximately 80% of patients with gMG are AChR autoantibody-positive (Ab+), while 5–8% are reported to have MuSK Ab+ gMG [2, 6]. Conventional treatments, such as corticosteroids and non-steroidal immunosuppressant therapy, aim to control symptoms rather than target the drivers of disease and are associated with adverse events and increased risk of infection, while treatment of exacerbations or refractory disease with intravenous immunoglobulin (IVIg) or plasma exchange (PLEX) requires prolonged administration under physician supervision [2, 7, 8].

The neonatal Fc receptor (FcRn) is a therapeutic target in gMG due to its role in extending the half-life of plasma immunoglobulin G (IgG), including pathogenic IgG autoantibodies, by salvaging and recycling IgG and preventing lysosomal degradation [9]. Rozanolixizumab is a humanized IgG4 monoclonal antibody that inhibits FcRn activity to prevent IgG recycling and reduce levels of IgG autoantibodies implicated in the pathogenesis of myasthenia gravis (MG) [9, 10]. In the randomized, double-blind, Phase 3, MycarinG study (NCT03971422; EudraCT 2019-000968-18), adult patients with AChR or MuSK Ab+ gMG were randomly assigned 1:1:1 to receive weekly subcutaneous infusions of rozanolixizumab 7 or 10 mg/kg or placebo for 6 weeks in addition to their current therapy [11]. Rozanolixizumab showed clinically meaningful improvements in patient-reported and investigator-assessed outcomes compared with placebo, including in MG Activities of Daily Living (MG-ADL), MG Composite (MGC) and Quantitative MG (QMG) total scores. Rozanolixizumab treatment was generally well tolerated after a single 6-week cycle. Rozanolixizumab is approved by the United States Food and Drug Administration for the treatment of adults with AChR or MuSK Ab+ gMG, with an initial 6-week cycle and subsequent treatment cycles based on clinical evaluation [12]. Rozanolixizumab is also approved in the European Union and the United Kingdom as an add-on to standard therapy for adults with AChR or MuSK Ab+ gMG, and in Japan for the treatment of gMG in adults who inadequately respond to steroids or other immunosuppressants [13, 14]. Further data were needed to evaluate the long-term safety and efficacy of rozanolixizumab treatment in patients with gMG. Here, we report findings from the Phase 3 open-label extension MG0004 study that investigated the long-term safety, tolerability and efficacy of up to 52 weekly rozanolixizumab infusions in adult patients with gMG. A plain-language summary of this study is available in Online Resource 1.

## Methods

### Study design and patients

MG0004 (NCT04124965; EudraCT 2019-000969-21) was a Phase 3, multicenter, randomized, open-label extension study that followed the double-blind, placebo-controlled MycarinG study. Full details of the MycarinG study design have been published previously [11]. In brief, eligible patients in MycarinG were aged ≥18 years, with AChR or MuSK Ab+ gMG (Myasthenia Gravis Foundation of America [MGFA] Class II–IVa), MG-ADL score ≥3 (for non-ocular symptoms), QMG score ≥11, and body weight ≥35 kg, and had been considered for treatment with additional therapy, such as IVIg or PLEX. Key exclusion criteria included severe oropharyngeal or respiratory muscle weakness (MGFA Class IVb) and total IgG concentration ≤5.5 g/L. Patients could enroll in MG0004 if they had either completed MycarinG or required rescue therapy during the MycarinG observation period and opted to enter MG0004 and receive rozanolixizumab in preference to receiving IVIg or PLEX. MG0004 was closed early in response to feedback from clinicians and patients regarding the burden of the study, particularly the requirement for patients to attend weekly visits to the study center for up to 52 weeks of treatment administration. Furthermore, chronic weekly dosing was not anticipated to be used in clinical practice. MG0007 (NCT04650854; EudraCT 2020-003230-20) was an open-label extension study of rozanolixizumab administered in 6-week treatment cycles driven by worsening of MG symptoms (Online Resource 2) that started after MG0004. After a minimum treatment duration of six visits, MG0004 patients could roll over into MG0007 when it opened for recruitment. Patients from MG0004 who had received ≥6 weekly doses of rozanolixizumab moved directly into the observation period of MG0007 once MG0007 had been initiated at their study center and MG0004 closed.

### Study treatment

In MG0004, patients from MycarinG were re-randomized 1:1 to receive once-weekly subcutaneous rozanolixizumab 7 or 10 mg/kg for up to 52 infusions, followed by an 8-week observation period after the last infusion. The final visit of MycarinG served as visit Week 1 in MG0004 and the maximum study duration was from visit Week 1 to visit Week 60. Rozanolixizumab was administered by subcutaneous infusion to the lower abdomen using a syringe pump. Patients could switch treatment dose at the investigators’ discretion; potential reasons for switching from 10 to 7 mg/kg included tolerability and toxicity, and worsening of efficacy or symptoms were reasons to switch from 7 to 10 mg/kg.

Rescue therapy with IVIg or PLEX was permitted for patients who experienced disease worsening, based on the investigator judgment. If a patient received rescue therapy during the study, treatment with rozanolixizumab was discontinued for a period of 2–6 weeks but visits continued, after which rozanolixizumab treatment could be recontinued at the investigators’ discretion.

### Outcomes and assessments

The primary objective of the MG0004 study was to evaluate the long-term safety and tolerability of chronic weekly rozanolixizumab in patients with gMG. The primary safety endpoints were the occurrence of treatment-emergent adverse events (TEAEs) and the occurrence of TEAEs leading to permanent discontinuation of study treatment. The secondary objective of the study was to evaluate the long-term efficacy of weekly rozanolixizumab treatment. Secondary efficacy endpoints included the change from baseline in MG-ADL, MGC, and QMG total scores and the use of rescue therapy during the study. Other endpoints included the change from baseline in MG Symptoms Patient-Reported Outcome (PRO) scale scores (Muscle Weakness Fatigability, Physical Fatigue and Bulbar Muscle Weakness), MG Impairment Index (MGII) and revised 15-item MG Quality of Life (MG-QoL 15r), pharmacodynamic analyses (total IgG, IgG subclasses and MG-specific autoantibodies) and immunogenicity analyses (incidence of anti-drug antibodies [ADAs] and neutralizing antibody status).

### Statistical analysis

All eligible patients from the MycarinG study were invited to participate in MG0004, and no formal sample size calculation was performed. Descriptive statistics included the number and percentage of patients for categorical variables, and mean or median data with measures of variation for continuous variables. Safety outcomes were assessed for patients who received ≥1 rozanolixizumab dose (safety set). TEAEs are reported per treatment group by the most recent dose received. Efficacy and pharmacodynamic outcomes are reported for the safety set by the first dose received. No statistical testing was performed for efficacy analyses. Baseline values were the last available values before or on the date of the first administration of rozanolixizumab in MG0004. Immunogenicity was assessed in all patients in the safety set with an evaluable baseline sample and ≥1 evaluable post-baseline value. Baseline values for ADAs were the last measurement before receiving the first rozanolixizumab infusion in MycarinG or MG0004. Statistical analysis was performed using SAS® Version 9.3. The study protocol and statistical analysis plan were published on ClinicalTrials.gov (NCT04124965) [15].

## Results

### Patient population and treatment exposure

**Fig. 1:**
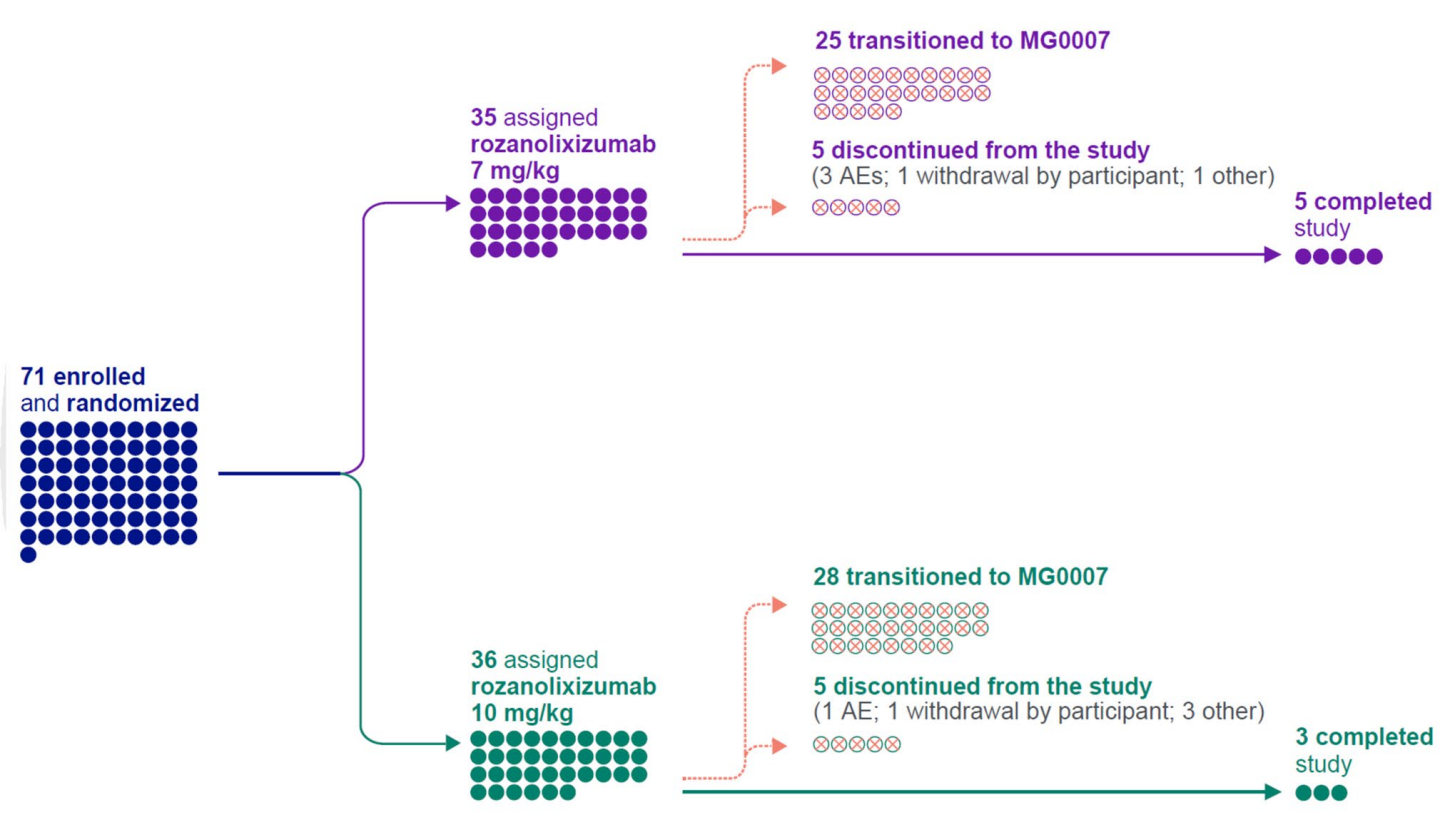
Patient flow. *AE* adverse event

Between October 29, 2019 and June 3, 2021, 71 patients entered MG0004 from the MycarinG study and were re-randomized to receive initial rozanolixizumab 7 mg/kg (*n* = 35) or rozanolixizumab 10 mg/kg (*n* = 36; Fig. 1). Patient flow from MycarinG to the MG0004 and MG0007 studies is detailed in Online Resource 2. In MG0004, one patient in the 10 mg/kg group was not treated with rozanolixizumab and was excluded from the safety set, resulting in 70 patients receiving ≥1 dose of rozanolixizumab. Demographics and baseline characteristics were generally balanced between the two rozanolixizumab dose groups, except that a higher proportion of patients in the 7 mg/kg group than in the 10 mg/kg group were treated in North America (Table 1). In addition, fewer patients in the 7 mg/kg group who required rescue therapy in the MycarinG observation period opted to instead enter MG0004 to receive rozanolixizumab compared with the 10 mg/kg group.

**Table 1:** Baseline demographics and patient characteristics (randomized set)

	Rozanolixizumab 7 mg/kg (*n* = 35)	Rozanolixizumab 10 mg/kg (*n* = 36)	Rozanolixizumab Total (*N* = 71)
Age, years, mean (SD)^a^	50.6 (14.2)	53.7 (17.2)	52.2 (15.8)
Sex, female, *n* (%)	19 (54.3)	19 (52.8)	38 (53.5)
Body weight, *n* (%)			
<50 kg	2 (5.7)	5 (13.9)	7 (9.9)
50 to <70 kg	8 (22.9)	5 (13.9)	13 (18.3)
70 to <100 kg	17 (48.6)	17 (47.2)	34 (47.9)
≥100 kg	8 (22.9)	9 (25.0)	17 (23.9)
Geographic region, *n* (%)			
North America	16 (45.7)	11 (30.6)	27 (38.0)
Europe	15 (42.9)	21 (58.3)	36 (50.7)
Asia (excl. Japan)	0	2 (5.6)	2 (2.8)
Japan	4 (11.4)	2 (5.6)	6 (8.5)
Race, *n* (%)			
Asian	4 (11.4)	4 (11.1)	8 (11.3)
Black	2 (5.7)	3 (8.3)	5 (7.0)
White	17 (48.6)	19 (52.8)	36 (50.7)
Missing^b^	12 (34.3)	10 (27.8)	22 (31.0)
Thymectomy, yes, *n* (%)^a^	14 (40.0)	15 (41.7)	29 (40.8)
AChR Ab+, *n* (%)	30 (85.7)	32 (88.9)	62 (87.3)
MuSK Ab+, *n* (%)	5 (14.3)	4 (11.1)	9 (12.7)
MG-ADL score, mean (SD)	8.4 (3.6)	8.4 (3.7)	8.4 (3.6)
QMG score, mean (SD)	15.2 (5.1)	15.4 (5.5)	15.3 (5.3)
Duration of disease, years, mean (SD)^a^	8.7 (9.7)	8.2 (8.4)	8.5 (9.0)
Prior gMG medication, *n* (%)			
Corticosteroids for systemic use	24 (68.6)	20 (55.6)	44 (62.0)
Immunosuppressants	19 (54.3)	18 (50.0)	37 (52.1)
Parasympathomimetics	30 (85.7)	30 (83.3)	60 (84.5)
Required rescue medication during MycarinG observation period before entering MG0004, *n* (%)	8 (22.9)	16 (44.4)	24 (33.8)

*Ab*+ autoantibody-positive, *AChR* acetylcholine receptor, *gMG* generalized myasthenia gravis, *MG-ADL* Myasthenia Gravis Activities of Daily Living, *MuSK* muscle-specific tyrosine kinase, *QMG* Quantitative Myasthenia Gravis, *SD* standard deviation

^a^Data obtained at MycarinG study baseline. ^b^Data on race were not permitted to be collected in France and Canada

An overall mean (standard deviation [SD]) treatment duration of 23.3 (14.5) weeks corresponded to 32.7 patient-years of rozanolixizumab treatment in MG0004. The mean (SD) duration of rozanolixizumab treatment was 22.9 (14.6) weeks in the 7 mg/kg group and 23.7 (14.6) weeks in the 10 mg/kg group. The mean (SD) number of infusions was 21.7 (13.0) and 21.6 (12.3), respectively. Over 50% of patients overall had ≥18 weekly infusions of rozanolixizumab in MG0004 (18 [51.4%] in the 7 mg/kg group and 20 [57.1%] in the 10 mg/kg group). After Week 6, patients could transition to the MG0007 study; 53 (74.6%) patients permanently discontinued MG0004 to transition to MG0007 once it was open. After Week 33, patient numbers were low (≤10 per treatment group at any scheduled assessment from Week 37). Eight (11.3%) patients received the maximum of 52 rozanolixizumab infusions in MG0004; seven of these patients subsequently enrolled in MG0007. There were a total of 32 dose changes during the treatment period. Excluding patients who switched for a single week, in the 7 mg/kg group, 5/35 patients switched to 10 mg/kg, of whom 3 stayed on the higher dose. In the 10 mg/kg group, 14/35 patients switched to 7 mg/kg, of whom 12 stayed on the lower dose.

### Safety

Overall, 60/70 (85.7%) patients who received ≥1 dose of rozanolixizumab reported TEAEs; 38/50 (76.0%) of these patients were in the 7 mg/kg group and 33/42 (78.6%) were in the 10 mg/kg group by most recent dose. Patients who switched doses may be counted in both treatment groups, hence the total of denominators in the two treatment groups is larger than the cohort of 70 patients. The most frequently reported TEAEs were headache, diarrhea, decreased blood IgG, nausea, pyrexia and urinary tract infection (Table 2).

**Table 2:** Overview of TEAEs (safety set)

	Rozanolixizumab 7 mg/kg (*n* = 50)^a^ *n* (%)	Rozanolixizumab 10 mg/kg (*n* = 42)^a^ *n* (%)	Rozanolixizumab total (*N* = 70) *n* (%)
Any TEAEs^b^	38 (76.0)	33 (78.6)	60 (85.7)
Headache	15 (30.0)	12 (28.6)	25 (35.7)
Diarrhea	6 (12.0)	7 (16.7)	13 (18.6)
Decreased blood IgG	6 (12.0)	5 (11.9)	11 (15.7)
Nausea	4 (8.0)	5 (11.9)	9 (12.9)
Pyrexia	4 (8.0)	3 (7.1)	7 (10.0)
UTI	5 (10.0)	2 (4.8)	7 (10.0)
Infections	13 (26.0)	9 (21.4)	20 (28.6)
Hypersensitivity reactions	7 (14.0)	5 (11.9)	12 (17.1)
Injection-site reactions	2 (4.0)	4 (9.5)	6 (8.6)
Serious TEAEs	7 (14.0)	2 (4.8)	9 (12.9)
Permanent discontinuation of study due to TEAEs	4 (8.0)	0	4 (5.7)
Congestive cardiac failure	1 (2.0)	0	1 (1.4)
Myasthenia gravis	3 (6.0)	0	3 (4.3)
TEAEs requiring dose change	0	1 (2.4)	1 (1.4)
Treatment-related TEAEs	25 (50.0)	18 (42.9)	41 (58.6)
Severe TEAEs^c^	12 (24.0)	5 (11.9)	17 (24.3)
Headache	3 (6.0)	2 (4.8)	5 (7.1)
Myasthenia gravis	2 (4.0)	1 (2.4)	3 (4.3)
All deaths (AEs leading to death)	0	0	0

Safety set by most recent dose

*AE* adverse event, *IgG* immunoglobulin G, *TEAE* treatment-emergent adverse event, *UTI* urinary tract infection

^a^Patients who switched doses are counted in both rozanolixizumab treatment groups but only once in the rozanolixizumab total group. ^b^Specific TEAEs listed are those occurring in ≥10% of patients overall. ^c^Specific severe TEAEs listed are those occurring in >1 patient overall

Three (6.0%) patients in the 7 mg/kg group and no patients in the 10 mg/kg group by most recent dose experienced TEAEs leading to discontinuation of rozanolixizumab and withdrawal from the study; two of these patients had TEAEs of MG worsening and 1 patient had congestive heart failure. One additional patient in the 7 mg/kg group had MG worsening while rozanolixizumab treatment was being withheld due to a low IgG level and withdrew from the study. Treatment-related TEAEs (per investigator assessment) were reported in 41/70 (58.6%) patients. Most TEAEs were of mild or moderate intensity, with severe TEAEs reported in 17/70 (24.3%) patients. The only severe TEAEs reported in >1 patient overall were headache and MG worsening (Table 2).

Serious TEAEs were reported in 9/70 (12.9%) patients. None of these serious TEAEs were considered by the investigator to be related to rozanolixizumab treatment. In the 7 mg/kg group, there were three serious TEAEs of MG worsening and one case each of abnormal kidney biopsy, congestive heart failure, muscular weakness and retinal detachment. One of the 3 patients with MG worsening and the patient with muscular weakness were in the observation period and not receiving rozanolixizumab at the time of the serious event. In the 10 mg/kg group, there was one serious TEAE each of pericarditis and MG worsening. There were no deaths reported during the study.

Infections were reported in 20/70 (28.6%) patients overall. There were no opportunistic, serious or severe infections, and no infections led to treatment discontinuation or study withdrawal. Hypersensitivity reactions were reported in 12/70 (17.1%) patients; none of these events led to treatment discontinuation or study withdrawal. Injection-site reactions were reported in 6/70 (8.6%) patients overall and none of the TEAEs by preferred term were reported in >1 patient in either dose group. There were no serious or severe events of hypersensitivity or injection-site reactions, and no anaphylactic reactions. No TEAEs related to albumin or lipid abnormalities were reported. Overall, mean vital sign measurements, electrocardiogram, hematology, clinical chemistry and urinalysis laboratory results remained stable over time. One patient became pregnant approximately 4 weeks after initiating treatment with rozanolixizumab 7 mg/kg, and treatment was permanently discontinued. The patient had a serious TEAE of muscular weakness 39 days after the last dose and received rescue therapy. At 38 weeks of gestation, the patient gave birth to a healthy baby with no complications.

### Efficacy

**Fig. 2:**
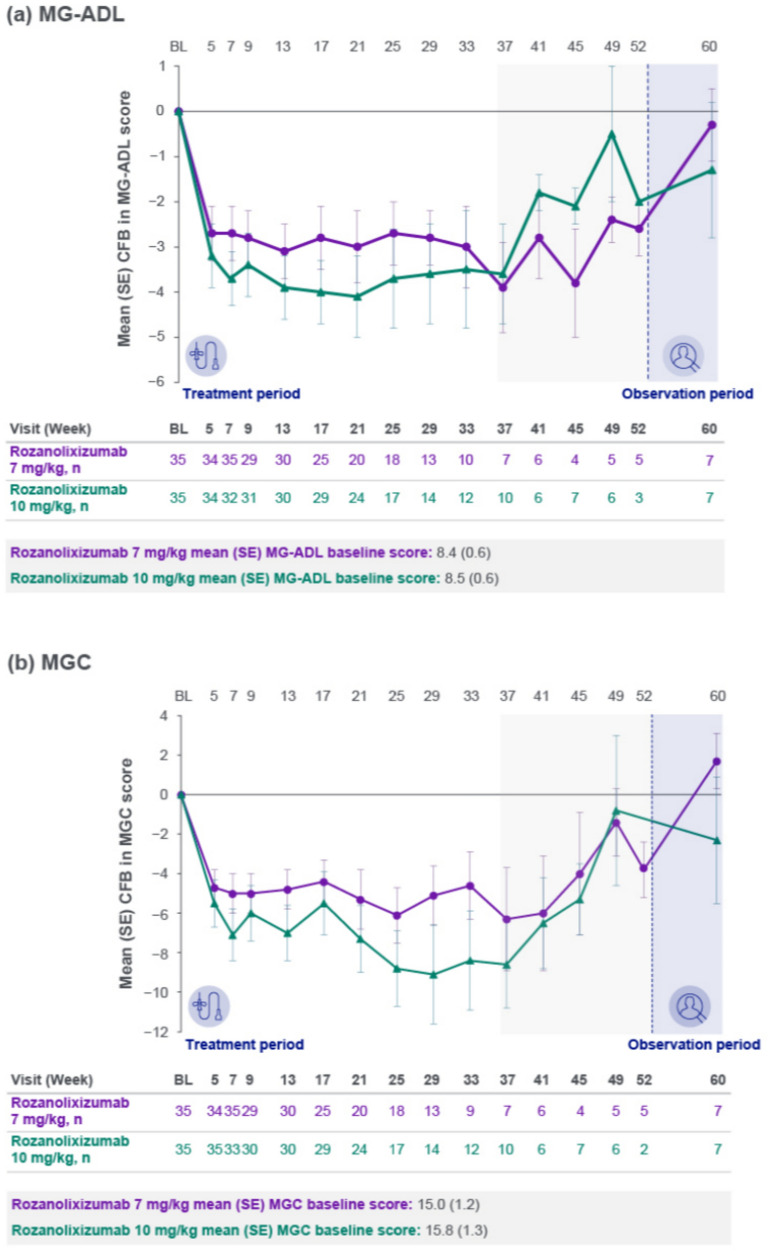

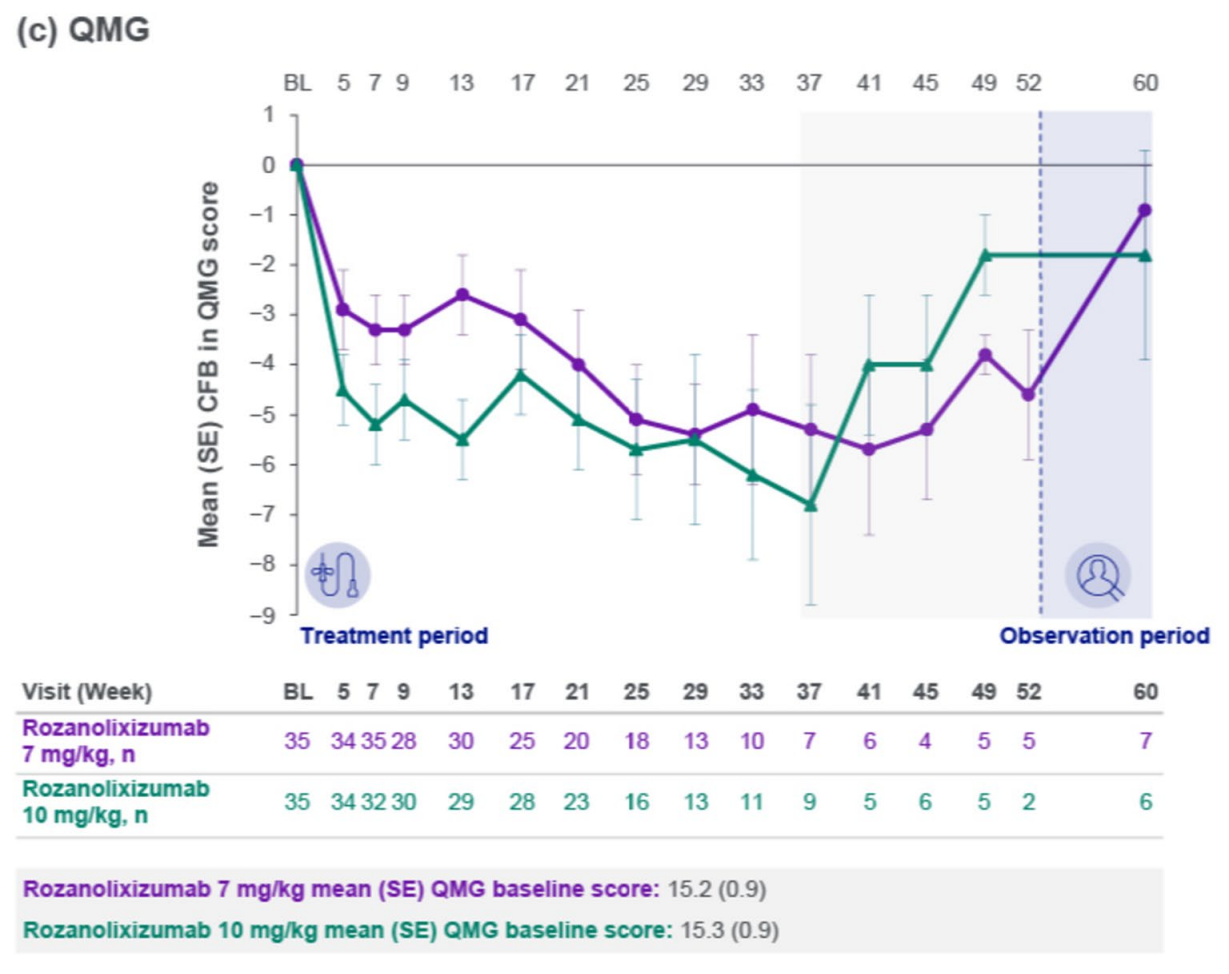
Mean change from baseline to Week 60 in **a** MG-ADL, **b** MGC and **c** QMG scores (safety set). Safety set by first dose received. The light gray area represents study visits at which patient numbers were low (≤10 per treatment group at any scheduled assessment). *BL* baseline**,** *CFB* change from baseline**,** *MG-ADL* Myasthenia Gravis Activities of Daily Living**,** *MGC* Myasthenia Gravis Composite**,** *QMG* Quantitative Myasthenia Gravis**,** *SE* standard error

Clinically meaningful improvements from MG0004 baseline in MG-ADL total score were observed in both rozanolixizumab dose groups (Fig. 2a), with a mean change from baseline consistently greater in the 10 mg/kg group compared with the 7 mg/kg group up to Week 33, after which patient numbers were low (≤10 per dose group at any scheduled assessment from Week 37). The maximum mean reduction from baseline up to Week 33 was 3.1 (Week 13) in the 7 mg/kg group and 4.1 (Week 21) in the 10 mg/kg group. A decrease from baseline in MG-ADL score was observed after 4 infusions (Week 5), the earliest time of assessment following treatment initiation, with a mean reduction from baseline of 2.7 and 3.2, respectively. After 6 infusions (Week 7), the mean reduction from baseline was 2.7 and 3.7, respectively. These changes were consistent with changes in MG-ADL score after 4 and 6 weekly infusions in the MycarinG study. Subgroups of patients with AChR Ab+ gMG and MuSK Ab+ gMG showed trends in MG-ADL score change from baseline (data not shown) that were similar to the overall population. Improvements from baseline in MG-ADL total score were generally consistent between other subgroups analyzed and the overall population, including by age, sex, MGFA disease class and baseline MG-ADL category (data not shown).

Similar trends were observed in MGC (Fig. 2b) and QMG (Fig. 2c) total scores. The maximum mean reduction from baseline in MGC up to Week 33 was 6.1 (Week 25) in the 7 mg/kg group and 9.1 (Week 29) in the 10 mg/kg group. The maximum mean reduction from baseline in QMG up to Week 33 was 5.4 (Week 29) and 6.2 (Week 33), respectively.

**Fig. 3:**
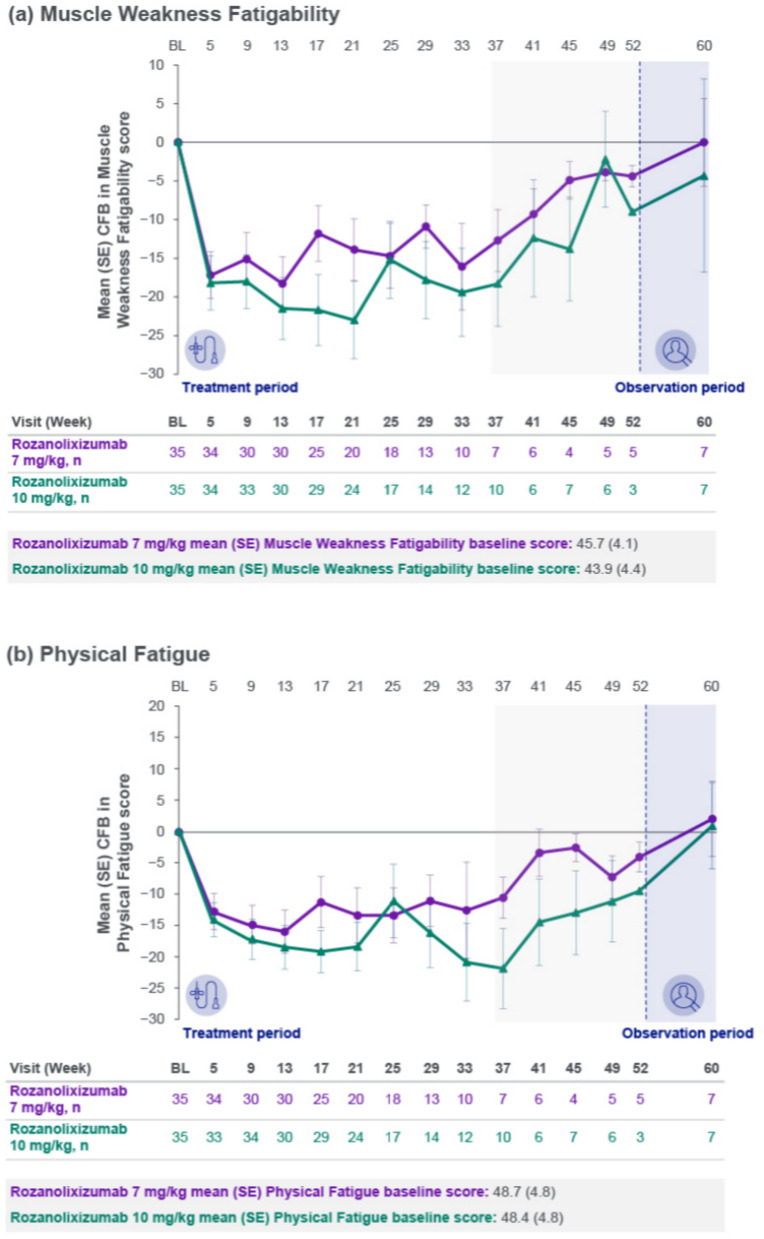

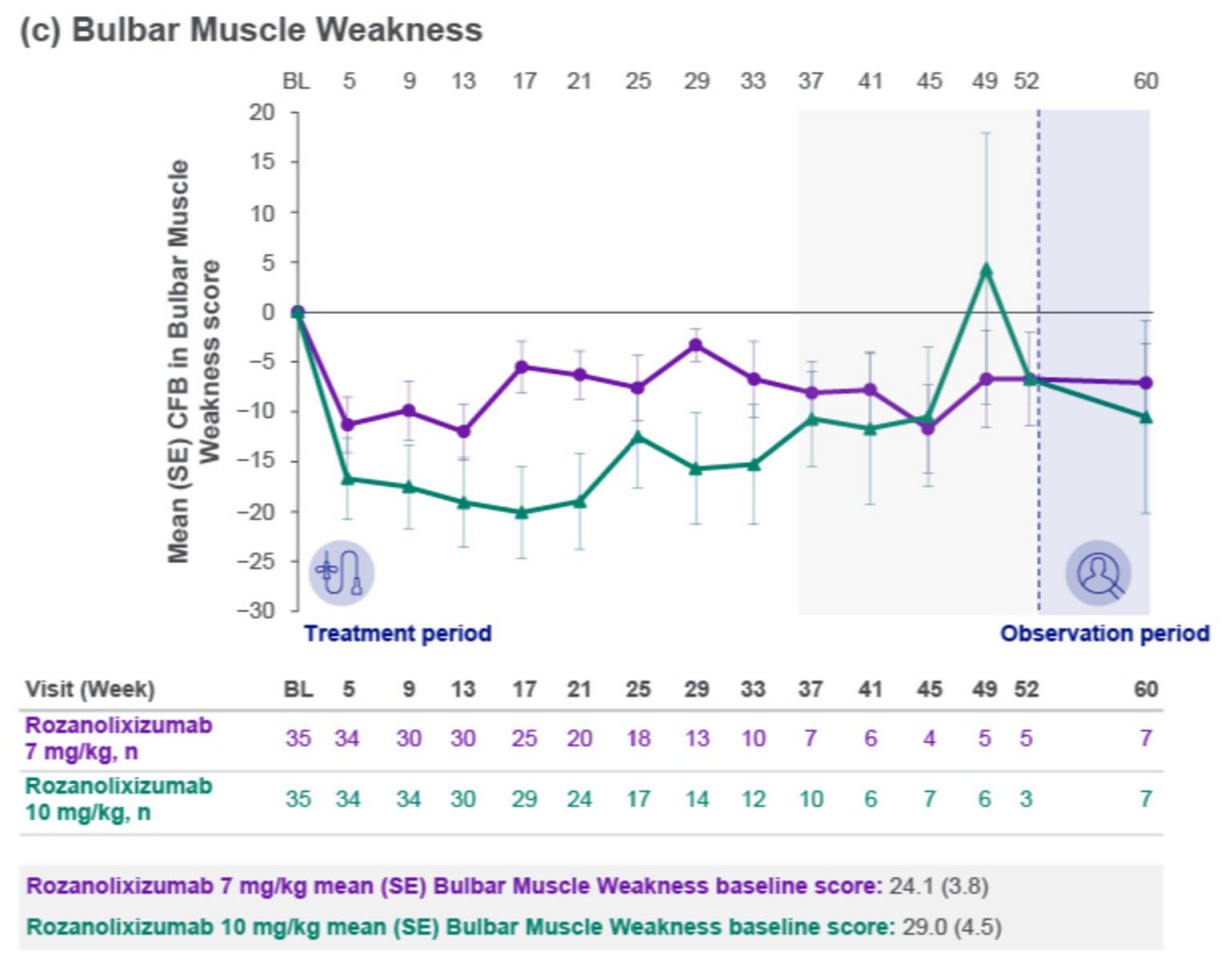
Mean change from baseline to Week 60 in MG Symptoms PRO scale scores: **a** Muscle Weakness Fatigability, **b** Physical Fatigue and **c** Bulbar Muscle Weakness (safety set). Safety set by first dose received. The light gray area represents study visits at which patient numbers were low (≤10 per treatment group at any scheduled assessment). *BL* baseline**,** *CFB* change from baseline**,** *MG* myasthenia gravis**,** *PRO* patient-reported outcome; SE, standard error

Improvements from baseline were observed in mean scores across the MG Symptoms PRO scales of Muscle Weakness Fatigability (Fig. 3a), Physical Fatigue (Fig. 3b) and Bulbar Muscle Weakness (Fig. 3c), with consistent trends up to Week 33. Reductions from baseline in MGII overall score, ocular subscore and generalized subscore were observed. Maximum mean reduction from baseline in MGII overall score up to Week 33 was 12.1 (Week 21, *n* = 15) in the 7 mg/kg group and 17.3 (Week 25, *n* = 16) in the 10 mg/kg group. Improvements from baseline were also observed in MG-QoL 15r scores, with a maximum mean reduction from baseline up to Week 33 of 5.1 (Week 21, *n* = 20) in the 7 mg/kg group and 6.5 (Week 33, *n* = 12) in the 10 mg/kg group.

Across the total 32.7 patient-years of rozanolixizumab treatment, rescue therapy was received by 4 (11.4%) patients in the 7 mg/kg group (2 each during the treatment and observation periods); all 4 patients received IVIg. Of those who received rescue therapy in the treatment period, 1 patient received 12 doses of rozanolixizumab 7 mg/kg followed by 8 doses at 10 mg/kg, and was treated with IVIg 6 days after the last dose; the patient discontinued MG0004 and enrolled in MG0007, initiating treatment at 10 mg/kg and receiving four symptom-driven cycles over 9 months before discontinuing MG0007 due to an adverse event of MG worsening. The second patient received 12 doses at 7 mg/kg and then switched to receive 1 dose at 10 mg/kg, and was treated with IVIg 5 days after the 10 mg/kg dose; the patient subsequently discontinued the study due to a TEAE of MG worsening.

### Pharmacodynamics and immunogenicity

**Fig. 4:**
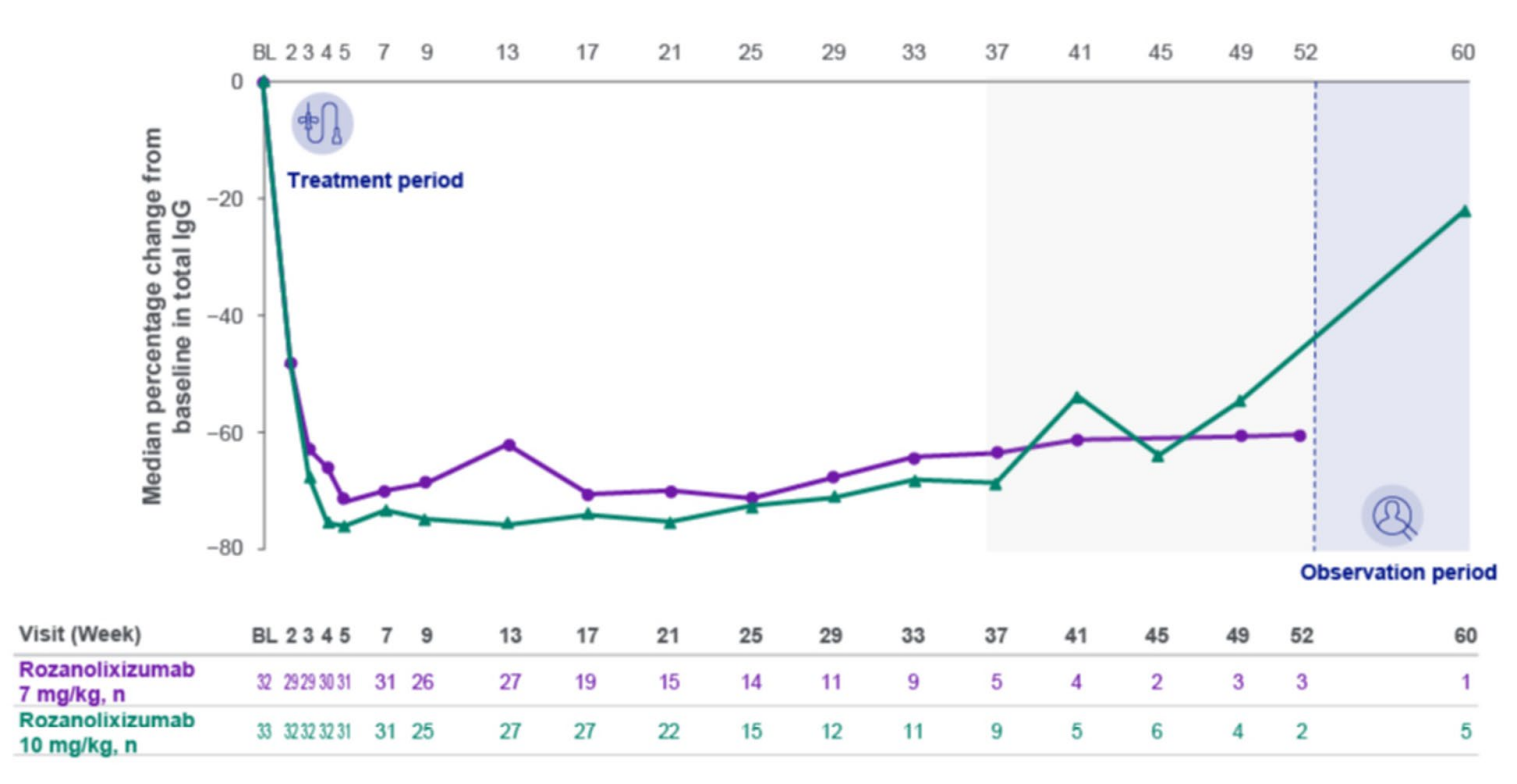
Median percentage change from baseline in total IgG (safety set). Safety set by the first dose received. Mean (SD) total IgG at baseline was 9.06 (3.16) g/L in the rozanolixizumab 7 mg/kg group and 8.78 (2.62) g/L in the rozanolixizumab 10 mg/kg group. The lowest mean (SD) total IgG during treatment was 2.55 (1.39) g/L at Week 17 in the rozanolixizumab 7 mg/kg group and 2.23 (1.00) g/L at Week 5 in the rozanolixizumab 10 mg/kg group. The light gray area represents study visits at which patient numbers were low (≤10 per treatment group at any scheduled assessment). *BL* baseline, *IgG* immunoglobulin G, *SD* standard deviation

A rapid median decrease from baseline in total IgG of 48.0% in the 7 mg/kg group and 47.9% in the 10 mg/kg group was observed at Week 2, the first on-treatment assessment in MG0004 (Fig. 4). Maximum reduction in total IgG was reached at approximately Week 5 and further reductions were not observed with continued weekly treatment. The median maximum reduction from baseline was 75.6% (*n* = 32) and 79.9% (*n* = 33), respectively. Similar trends were observed across all IgG subclasses, with a median maximum reduction from baseline ranging from 70.8% to 81.6% in the 7 mg/kg group and 62.5–83.1% in the 10 mg/kg group. There was also a rapid decrease from baseline in AChR antibody (Ab) levels; while data were only available for 3 patients in each group at the first assessment (Week 5), at Week 9 the median reduction was 60.1% in the 7 mg/kg group (*n* = 18) and 61.5% in the 10 mg/kg group (*n* = 17). Time profiles for total IgG and AChR Ab levels were consistent for both rozanolixizumab dose groups.

All patients tested (*n* = 69) were ADA-negative at the MG0004 study baseline. Overall, 37 (53.6%) patients developed treatment-emergent ADAs to rozanolixizumab, of whom 18 developed neutralizing ADAs (26.1%). For most patients with ADAs, the first occurrence of treatment-emergent ADA positivity was at Week 5 (14 [20.3%] patients) or Week 9 (6 [9.8%] patients), the first two time points for ADA assessment, and only 7 patients had their first treatment-emergent ADA positivity at a later assessment. Patterns were similar between patients who received rozanolixizumab and those who received placebo in the MycarinG study. In general, the presence of treatment-emergent or neutralizing ADAs did not have a clinically meaningful effect on efficacy (MG-ADL total score) or pharmacodynamics (total IgG levels). No trends were observed in the safety profile of rozanolixizumab based on ADA status.

## Discussion

MG0004 was an open-label study, with a treatment duration of up to 52 weekly infusions, that assessed the long-term safety and tolerability of chronic rozanolixizumab treatment. Long-term weekly rozanolixizumab was generally well tolerated, with a safety profile consistent with that reported in MycarinG [11] and similar to that with repeated cycles of rozanolixizumab treatment in MG0007 [16]. Two patients had TEAEs of MG worsening which led to the discontinuation of rozanolixizumab. TEAEs were mostly mild or moderate and chronic weekly treatment did not increase the risk of headache or infections compared with one cycle in MycarinG, in line with results observed with repeated symptom-driven treatment cycles. Some differences in the incidence of TEAEs between the dose groups were observed, but as events were reported by the most recent dose received it is difficult to compare the two groups, and no clear underlying reasons for the differences were identified. The only serious TEAE reported in >1 patient was MG worsening, reported in 4 patients; additionally, 1 patient experienced a serious TEAE coded as muscular weakness.

Most cases of headache were mild or moderate and there were no serious TEAEs or permanent discontinuations of treatment due to headache. Five severe headaches were reported during the MG0004 study in 3 patients who had received placebo in the MycarinG study and 1 patient each who had previously received rozanolixizumab 7 and 10 mg/kg. One patient who experienced a severe headache in MG0004 also reported a severe headache in MycarinG but continued rozanolixizumab treatment in both studies. All severe headache events occurred early during treatment (2–3 days after the first dose) and, similar to experiences in MycarinG, were mostly managed with over-the-counter medication, with all patients fully recovered with no sequelae. The mechanism by which these headaches occur is unknown. Decreased blood IgG was expected based on the mechanism of action of rozanolixizumab [9], and all TEAEs of this type were mild or moderate. As with other immunomodulatory therapies, there is a concern of increased risk of infection with FcRn inhibitors [17], and individuals with MG may be at greater risk of infection compared with the general population [18]. In MG0004, the rate of infections was 26.0% in the 7 mg/kg group and 21.4% in the 10 mg/kg group, similar to that reported in rozanolixizumab-treated patients in MycarinG and slightly higher than 19.4% reported in placebo-treated patients. No opportunistic, serious or severe infections were reported. Most infections were considered by the investigator to be unrelated to rozanolixizumab treatment. Subcutaneous infusions of rozanolixizumab were generally well tolerated with a low incidence of local injection-site reactions, and no serious or severe hypersensitivity reactions or anaphylactic reactions were reported. There was no clinically relevant impact on albumin or lipid levels.

An analysis of the safety and tolerability of cyclic treatment in MycarinG and MG0007 (up to an interim data cut-off of July 8, 2022) represented 121.1 patient-years’ exposure to rozanolixizumab treatment in patients with ≥1 year of study participation [16]. Consistent with MG0004, the majority of TEAEs were mild to moderate in intensity, and incidence did not increase with repeated cyclic treatment across the MycarinG and MG0007 studies. The analysis, together with the results of this study, provides insight into the long-term safety and tolerability of rozanolixizumab treatment. There are limited data on the safety of rozanolixizumab in patients with short intervals between treatment cycles; a pooled analysis of efficacy across MycarinG, the first 6 weeks of MG0004, and MG0007 interim data found that approximately 10% of patients had a treatment-free interval of <4 weeks [19]. The safety profile of chronic weekly treatment in MG0004 suggests that rozanolixizumab is expected to be generally well tolerated in patients who may require frequent treatment cycles based on clinical evaluation of symptoms.

Clinically meaningful mean improvements from the MG0004 baseline were observed with rozanolixizumab treatment during the first 7 weeks across the three MG-specific secondary efficacy endpoints, consistent with results from the 6-week treatment cycle in MycarinG. Mean reduction from baseline in MG-ADL total score at Week 7 was 2.7 in the 7 mg/kg group and 3.7 in the 10 mg/kg group, comparable with least squares mean reduction from baseline at Day 43 of 3.37 and 3.40, respectively, in the MycarinG study [11]. The improvement was maintained long term in MG0004. Improvements from baseline in MGC and QMG total scores were also similar to those observed in the double-blind study. Across the total 32.7 patient-years of rozanolixizumab treatment, only 2 patients received rescue therapy during the treatment period. Improvements from baseline were also observed in three MG Symptoms PRO scales: Muscle Weakness Fatigability, Physical Fatigue and Bulbar Muscle Weakness. The use of MG Symptoms PRO in MG0004 enabled a granular assessment of treatment effect across the different muscle groups affected by gMG, and included evaluation of physical fatigue, which is not included in MG-ADL and has been identified by patients as an important set of symptoms [5, 20–22]. Improvements with rozanolixizumab treatment were also consistent across the other PRO instruments utilized in the study, MGII and MG-QoL 15r. All efficacy outcomes showed stable trends until Week 33, after which there were ≤10 patients per dose group at any scheduled assessment. Once the MG0007 study was open in a patient’s region, patients were permitted to switch from MG0004 to receive symptom-driven cyclical treatment with rozanolixizumab instead of chronic weekly treatment; a limitation of this study is that the number of study participants in MG0004 decreased steadily after Week 22 due to rollover into MG0007.

Rapid reductions were observed in total IgG and AChR Abs for both dose groups, within the range associated with clinical benefit and consistent with observations in MycarinG [11]. Reduction in IgG levels can vary in individual patients, and at the individual level IgG monitoring could be considered in alignment with local guidelines and clinical practice. For both rozanolixizumab groups, total serum IgG levels, AChR Ab levels, and scores for MG-ADL, MGC and QMG had consistent time profiles. Similar to results from MycarinG, the development of treatment-emergent ADAs or neutralizing ADAs did not have a clinically meaningful impact on the pharmacodynamic response (based on total IgG levels) or efficacy (based on MG-ADL total score) of rozanolixizumab, and no trends were observed in the safety profile based on ADA category.

Overall, results from the MG0004 open-label extension study indicate that chronic weekly administration of rozanolixizumab was generally well tolerated, and efficacy was maintained with long-term treatment in patients with AChR Ab+ or MuSK Ab+ gMG. While rozanolixizumab is approved for cyclical treatment based on clinical evaluation of symptoms, these data provide support for the long-term safety, tolerability and efficacy of rozanolixizumab in patients who may require frequent cycles with short treatment-free intervals as determined by their treating physician.

## Supplementary Information

Below is the link to the electronic supplementary material.

Supplementary file1 (PDF 2832 KB)

## Data Availability

Underlying data from this manuscript may be requested by qualified researchers 6 months after product approval in the US and/or Europe, or global development is discontinued, and 18 months after trial completion. Investigators may request access to anonymized individual patient-level data and redacted trial documents which may include: analysis-ready datasets, study protocol, annotated case report form, statistical analysis plan, dataset specifications and clinical study report. Prior to the use of the data, proposals need to be approved by an independent review panel at www.vivli.org and a signed data-sharing agreement will need to be executed. All documents are available in English only, for a pre-specified time, typically 12 months, on a password-protected portal.
