# Supplementary material for: Safety and efficacy of chronic weekly rozanolixizumab in generalized myasthenia gravis: the randomized open-label extension MG0004 study
Source: J Neurol. 2025 Mar 19;272(4):275. doi: 10.1007/s00415-025-12958-9 (PMC11923026; doi:10.1007/s00415-025-12958-9)
Supplement: Supplementary file 1 — Supplementary file1 (PDF 2832 KB) [file 415_2025_12958_MOESM1_ESM.pdf]

Supplementary Information

Safety and efficacy of chronic weekly rozanolixizumab in generalized myasthenia gravis: The randomized open-label extension MG0004 study

Vera Bril[1], Artur Drużdż[2], Julian Grosskreutz[3], Ali A. Habib[4], Henry J. Kaminski[5], Renato Mantegazza[6], Sabrina Sacconi[7], Kimiaki Utsugisawa[8], Tuan Vu[9], Marion Boehnlein[10], Maryam Gayfieva[11], Bernhard Greve[10], Franz Woltering[10], John Vissing[12] on behalf of the MG0004 study investigators

[1]Ellen and Martin Prosserman Centre for Neuromuscular Diseases, Toronto General Hospital, University of Toronto, Toronto, Ontario, Canada; [2]Department of Neurology, Municipal Hospital, Poznań, Poland; [3]Precision Neurology of Neuromuscular Diseases, Department of Neurology, University of Lübeck, Lübeck, Germany; [4]MDA ALS & Neuromuscular Center, Department of Neurology, University of California, Irvine, Orange, CA, USA; [5]Department of Neurology & Rehabilitation Medicine, George Washington University, Washington, DC, USA; [6]Emeritus and Past Director, Department of Neuroimmunology and Neuromuscular Diseases, Fondazione IRCCS, Istituto Nazionale Neurologico Carlo Besta, Milan, Italy; [7]Université Côte d'Azur, Peripheral Nervous System & Muscle Department, Pasteur 2 Hospital, Centre Hospitalier Universitaire de Nice, Nice, France; [8]Department of Neurology, Hanamaki General Hospital, Hanamaki, Japan; [9]Department of Neurology, University of South Florida Morsani College of Medicine, Tampa, FL, USA; [10]UCB, Monheim, Germany; [11]UCB, Slough, UK; [12]Copenhagen Neuromuscular Center, Department of Neurology, Rigshospitalet, University of Copenhagen, Copenhagen, Denmark

Journal: Journal of Neurology

Corresponding author: Dr Vera Bril

Email: vera.bril@utoronto.ca

Table of Contents

Supplementary Information 3

Online Resource 1 Plain language summary 3

Online Resource 2 Patient flow from MycarinG through MG0004 and MG0007 4

Online Resource 3 MG0004 co-investigators and contributors 5

Supplementary Information

Online Resource 1 Plain language summary

Generalized myasthenia gravis (gMG) is a neuromuscular disease that causes unpredictable muscle weakness which may require lifelong treatment. A Phase 3 study called MycarinG (NCT03971422) showed that one 6-week cycle of once-weekly rozanolixizumab treatment improved disease symptoms compared with placebo in adult patients with gMG. After completing the MycarinG study, patients could enroll in the open-label extension study MG0004 (NCT04124965), which assessed the safety and efficacy of long-term weekly rozanolixizumab treatment. Patients received once-weekly rozanolixizumab 7 mg/kg or 10 mg/kg for up to 52 weeks, followed by an 8-week observation period.

In the MG0004 study, 70 patients received rozanolixizumab 7 mg/kg (n=35) or 10 mg/kg (n=35). Mean treatment duration was 22.9 and 23.7 weeks, respectively; study participation was low after Week 33 because patients had the option to enroll in a different open-label extension study to receive symptom-driven cycles of rozanolixizumab instead of continuous weekly treatment. In MG0004, side effects were reported in 85.7% of patients. Most side effects were mild or moderate. The most common side effects were headache (35.7% of patients), diarrhea (18.6% of patients) and decreased blood immunoglobulin G (15.7% of patients). There were no reports of any opportunistic infections, serious or severe infections, serious or severe hypersensitivity reactions, serious or severe injection-site reactions, any anaphylactic reactions or any side effects related to albumin or lipid abnormalities. Improvements from baseline in the Myasthenia Gravis Activities of Daily Living score were observed; the largest mean reduction from baseline was 3.1 in the 7 mg/kg group at Week 13 and 4.1 in the 10 mg/kg group at Week 21.

In this study, treatment with up to 52 weekly doses of rozanolixizumab was generally well tolerated and improvements across disease-specific outcomes were maintained, supporting the long-term use of rozanolixizumab for the treatment of patients with gMG.

Online Resource 2 Patient flow from MycarinG through MG0004 and MG0007

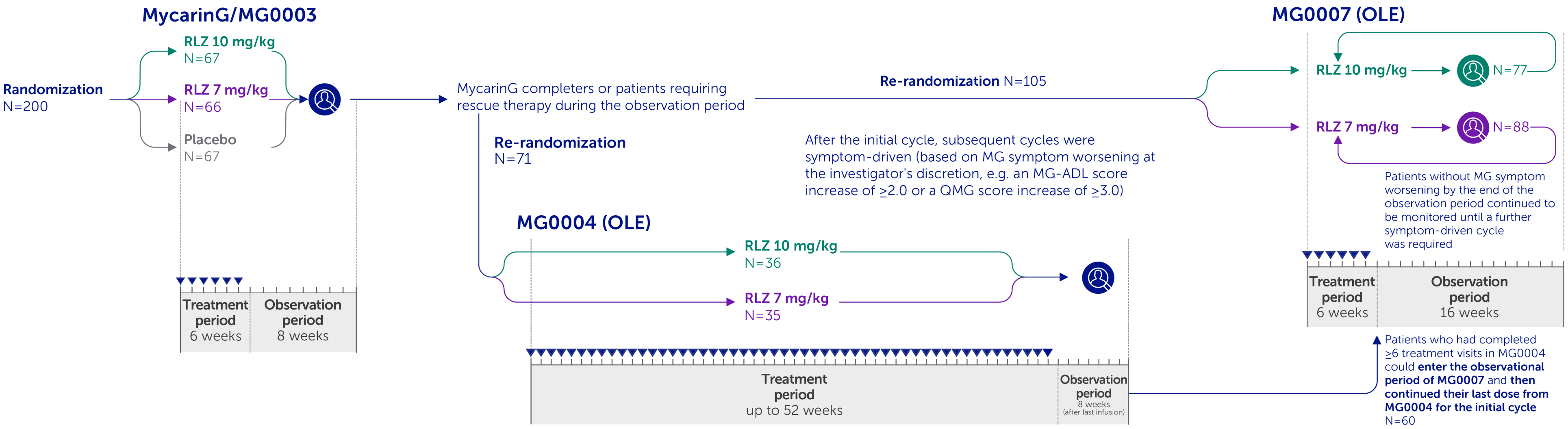

Figure adapted with permission from Bril V, Drużdż A, Grosskreutz J, Habib AA, Mantegazza R, Sacconi S, Utsugisawa K, et al (2025) Rozanolixizumab in generalized myasthenia gravis: Pooled analysis of the Phase 3 MycarinG study and two open-label extensions. J Neuromuscul Dis :22143602241305511. https://doi.org/10.1177/22143602241305511. MG, myasthenia gravis; MG-ADL, Myasthenia Gravis Activities of Daily Living; OLE, open-label extension; QMG, Quantitative Myasthenia Gravis; RLZ, rozanolixizumab.

Online Resource 3 MG0004 co-investigators and contributors

Name	Location	Role	Contribution
**Rodrigo Álvarez-Velasco**	Hospital de la Santa Creu i Sant Pau, Barcelona, Spain	Principal Investigator	Supported with the acquisition of data
**Radwa Aly**	Department of Neurology & Rehabilitation Medicine, George Washington University, Washington, DC, USA	Study Coordinator	Supported with the acquisition of data
**Henning Andersen**	Department of Neurology, Aarhus University Hospital, Aarhus, Denmark	Principal Investigator	Supported with the acquisition of data
**Giovanni Antonini**	Sapienza University of Rome, Department of Neurosciences, Mental Health and Sensory Organs (NESMOS), Italy	Principal Investigator	Supported with the acquisition of data
**Aramide Balogun**	Houston Methodist Hospital, Houston, TX, USA	Study Coordinator	Supported with the acquisition of data
**Ruggero Barnabei**	Fondazione Mondino Istituto Neurologico Nazionale a Carattere Scientifico – IRCCS, Pavia, Italy	Sub Investigator	Supported with the acquisition of data
**Said Beydoun**	University of Southern California Keck School of Medicine, Los Angeles, CA, USA	Principal Investigator	Supported with the acquisition of data
**Franz Blaes**	Klinik für Neurologie, Klinikum Oberberg, Gummersbach, Germany	Principal Investigator	Supported with the acquisition of data
**Silvia Bonarino**	Fondazione Istituto di Ricovero e Cura a Carattere Scientifico, Istituto Neurologico Carlo Besta, Italy	Sub Investigator	Supported with the acquisition of data
**Anna Boss Soevang**	Rigshospitalet, Denmark	Study Coordinator	Supported with the acquisition of data
**Nazibrola Botchorishvili**	LTD Simon Khechinashvili University Hospital, Georgia	Sub Investigator	Supported with the acquisition of data
**Stephan A. Botez**	Centre Hospitalier de l'Université de Montréal, Montreal, QC, Canada	Principal Investigator	Supported with the acquisition of data
**Ivo Bozovic**	Neurology Clinic, University Clinical Center of Serbia, Faculty of Medicine, University of Belgrade, Belgrade, Serbia	Sub Investigator	Supported with the acquisition of data
**Paulina Budzinska**	Miejskie Centrum Medyczne JONSCHER im. dr Karola Jonschera w Łodzi, Lodz, Poland	Sub Investigator	Supported with the acquisition of data
**Pietro Businaro**	Fondazione Mondino Istituto Neurologico Nazionale a Carattere Scientifico – IRCCS, Pavia, Italy	Sub Investigator	Supported with the acquisition of data

**Lucia Campetella**	PU A. Gemelli, Università Cattolica del Sacro Cuore, Italy	Sub Investigator	Supported with the acquisition of data
**Ana Belen Cánovas**	Hospital Universitari Vall D Hebron, Spain	Sub Investigator	Supported with the acquisition of data
**Carlos Casasnovas**	Hospital Universitari de Bellvitge-IDIBELL and CIBERER, Barcelona, Spain	Principal Investigator	Supported with the acquisition of data
**Hou-Chang Chiu**	Department of Neurology, Shin Kong Wu Ho-Su Memorial Hospital, Taipei, Taiwan	Sub Investigator	Supported with the acquisition of data
**His-Chieh Chou**	Taipei Veterans General Hospital, Taipei, Taiwan	Study Coordinator	Supported with the acquisition of data
**Adam Comer**	Indiana University Health Neuroscience Center, Indianapolis, IN, USA	Sub Investigator	Supported with the acquisition of data
**Elena Cortés Vicente**	Hospital de la Santa Creu i Sant Pau, Barcelona, Spain	Principal Investigator	Supported with the acquisition of data
**Roberto D'Angelo**	IRCCS Istituto delle Scienze Neurologiche di Bologna, Bologna, Italy	Sub Investigator	Supported with the acquisition of data
**Lubna Daniyal**	University Health Network, Toronto, ON, Canada	Study Coordinator	Supported with the acquisition of data
**Annie Dionne**	CHU de Québec – Université Laval, Hôpital Enfant-Jésus, Québec, QC, Canada	Principal Investigator	Supported with the acquisition of data
**Péter Diószeghy**	Szabolcs-Szatmár-Bereg County Hospitals and University Teaching Hospital, Nyiregyháza, Hungary	Principal Investigator	Supported with the acquisition of data
**Laura Fionda**	AOU Sant’Andrea Roma, Italy	Sub Investigator	Supported with the acquisition of data
**Denis Flemm**	UCB, Monheim, Germany	Operational study conduct	Supported with the acquisition of data
**Rita Frangiamore**	Fondazione Istituto di Ricovero e Cura a Carattere Scientifico, Istituto Neurologico Carlo Besta, Italy	Sub Investigator	Supported with the acquisition of data
**Manuela Gambella**	Université Côte d’Azur, Peripheral Nervous System and Muscle Department, Pasteur 2 Hospital, Centre Hospitalier Universitaire de Nice, Nice, France	Study Coordinator	Supported with the acquisition of data
**Rachana K. Gandhi Mehta**	Atrium Health Wake Forest Baptist, Winston-Salem, NC, USA	Principal Investigator	Supported with the acquisition of data
**Matteo Garibaldi**	AO S. Andrea, Università degli Studi di Roma La Sapienza, Italy	Sub Investigator	Supported with the acquisition of data

**Matteo Gastaldi**	Fondazione Mondino Istituto Neurologico Nazionale a Carattere Scientifico – IRCCS, Pavia, Italy	Principal Investigator	Supported with the acquisition of data
**Christian Geis**	Klinik für Neurologie, Universtitätsklinikum Jena, Jena, Germany	Principal Investigator	Supported with the acquisition of data
**Hannah George**	University of California, San Francisco Medical Center, San Francisco, CA, USA	Study Coordinator	Supported with the acquisition of data
**Stefan Gingele**	Medizinische Hochschule Hannover, Germany	Sub Investigator	Supported with the acquisition of data
**Monica Grau Martin**	Hospital Universitario de Bellvitge, Spain	Study Coordinator	Supported with the acquisition of data
**Yuh-Cherng Guo**	China Medical University Hospital, Taichung, Taiwan	Principal Investigator	Supported with the acquisition of data
**Gerardo Gutiérrez Gutiérrez**	Neurology Department, Hospital Universitario Infanta Sofía. Universidad Europea de Madrid. Department. of Medicine, Faculty of Biomedical and Health Sciences. Madrid, Spain	Principal Investigator	Supported with the acquisition of data
**Francesco Habetswallner**	Clinical Neurophysiology Unit, Cardarelli Hospital, Naples, Italy	Principal Investigator	Supported with the acquisition of data
**Lina Hassoun**	Klinik für Neurologie, Universitätsmedizin Göttingen, Göttingen, Germany	Sub Investigator	Supported with the acquisition of data
**Sonja Holm-Yildiz**	Copenhagen Neuromuscular Center, Department of Neurology, Rigshospitalet, University of Copenhagen, Copenhagen, Denmark	Sub Investigator	Supported with the acquisition of data
**Faraz Hussain**	University of Alberta, Edmonton, AB, Canada	Study Coordinator	Supported with the acquisition of data
**Francisca Iniesta**	Multiple Sclerosis and Clinical Neuroimmunology Unit, Neurology Department, Hospital Clínico Universitario Virgen de la Arrixaca, Murcia, Spain	Study Coordinator	Supported with the acquisition of data
**Viktoriya Irodenko**	University of California, San Francisco Medical Center, San Francisco, CA, USA	Sub Investigator	Supported with the acquisition of data
**Marina Janelidze**	Simon Khechinashvili University Hospital, Tbilisi, Georgia	Principal Investigator	Supported with the acquisition of data
**Min Kang**	University of California, San Francisco Medical Center, San Francisco, CA, USA	Sub Investigator	Supported with the acquisition of data
**Chafic Karam**	University of Pennsylvania, Philadelphia, PA, USA	Principal Investigator	Supported with the acquisition of data

**Denis Korobko**	State Novosibirsk Regional Clinical Hospital, Novosibirsk, Russia	Sub Investigator	Supported with the acquisition of data
**Sergey Kotov**	Vladimirsky Moscow Regional Research and Clinical Institute, Moscow, Russia	Principal Investigator	Supported with the acquisition of data
**Michal Kretkowski**	Clinical Research Center Sp. Z o.o., Medic-R Sp. K., Poland	Sub Investigator	Supported with the acquisition of data
**Nana Kvirkvelia**	LTD Petre Sarajishvili Institute of Neurology, Georgia	Sub Investigator	Supported with the acquisition of data
**Antonio Lauletta**	AO S. Andrea, Università degli Studi di Roma La Sapienza, Italy	Sub Investigator	Supported with the acquisition of data
**Yi-Chung Lee**	Taipei Veterans General Hospital, Taipei, Taiwan	Principal Investigator	Supported with the acquisition of data
**Luca Leonardi**	AOU Sant'Andrea Roma, Italy	Sub Investigator	Supported with the acquisition of data
**Kore Liow**	Hawaii Pacific Neuroscience, Honolulu, HI, USA	Principal Investigator	Supported with the acquisition of data
**Arnau Llauradó Gayete**	Hospital Universitari Vall D Hebron, Spain	Sub Investigator	Supported with the acquisition of data
**Sara Llufriu**	Servicio de Neurología ICN, Hospital Clinic de Barcelona, Universitat de Barcelona, Barcelona, Spain	Sub Investigator	Supported with the acquisition of data
**Catherine Lomen-Hoerth**	University of California, San Francisco Medical Center, San Francisco, CA, USA	Principal Investigator	Supported with the acquisition of data
**Jan D. Lünemann**	Klinik für Neurologie, Universitätsklinikum Münster, Münster, Germany	Sub Investigator	Supported with the acquisition of data
**Lorenzo Maggi**	Fondazione Istituto di Ricovero e Cura a Carattere Scientifico, Istituto Neurologico Carlo Besta, Italy	Sub Investigator	Supported with the acquisition of data
**Eugenia Martínez Hernández**	Servicio de Neurología ICN, Hospital Clinic de Barcelona, Universitat de Barcelona, Barcelona, Spain	Principal Investigator	Supported with the acquisition of data
**Gianvito Masi**	PU A. Gemelli, Università Cattolica del Sacro Cuore, Italy	Sub Investigator	Supported with the acquisition of data
**Marion Masingue**	Neurology Department, Pitié Salpêtrière-Charles Foix Hospital Group, AP-HP, Sorbonne University, Paris, France	Sub Investigator	Supported with the acquisition of data
**Rami Massie**	Clinical Research Unit, The Montreal Neurological Institute, Montreal, QC, Canada	Sub Investigator	Supported with the acquisition of data

**Marco Masullo**	Istituto delle Scienze Neurologiche, Italy	Sub Investigator	Supported with the acquisition of data
**Federico Mazzacane**	Fondazione Mondino Istituto Neurologico Nazionale a Carattere Scientifico – IRCCS, Pavia, Italy	Sub Investigator	Supported with the acquisition of data
**Nora Möhn**	Medizinische Hochschule Hannover, Germany	Sub Investigator	Supported with the acquisition of data
**Stefania Morino**	AOU Sant'Andrea Roma, Italy	Sub Investigator	Supported with the acquisition of data
**Kelsey Moulton**	University of Pennsylvania, Philadelphia, PA, USA	Study Coordinator	Supported with the acquisition of data
**Tahseen Mozaffar**	MDA ALS and Neuromuscular Center, University of California, Irvine, CA, USA	Sub Investigator	Supported with the acquisition of data
**Elene Nebadze**	LTD Petre Sarajishvili Institute of Neurology, Georgia	Sub Investigator	Supported with the acquisition of data
**Velina Nedkova-Hristova**	Hospital Universitari de Bellvitge-IDIBELL and CIBERER, Barcelona, Spain	Sub Investigator	Supported with the acquisition of data
**Eduardo Ng**	University Health Network, Toronto, ON, Canada	Study Coordinator	Supported with the acquisition of data
**Ekaterina Novikova**	Vladimirsky Moscow Regional Research and Clinical Institute, Moscow, Russia	Sub Investigator	Supported with the acquisition of data
**Izabella Obál**	Department of Neurology, Aalborg University Hospital, Aalborg, Denmark	Principal Investigator	Supported with the acquisition of data
**Anita Palsgård**	Aalborg Sygehus Nord, Denmark	Study Coordinator	Supported with the acquisition of data
**Claudia Papi**	PU A. Gemelli, Università Cattolica del Sacro Cuore, Italy	Sub Investigator	Supported with the acquisition of data
**Lorena Pérez**	Hospital Universitario Infanta Sofía, Spain	Study Coordinator	Supported with the acquisition of data
**Stojan Peric**	Neurology Clinic, University Clinical Center of Serbia, Faculty of Medicine, University of Belgrade, Belgrade, Serbia	Principal Investigator	Supported with the acquisition of data
**Mikhail Petrov**	St. Petersburg Regional Clinical Hospital, Russia	Sub Investigator	Supported with the acquisition of data
**Nicolai Rasmus Preisler**	Rigshospitalet, Denmark	Sub Investigator	Supported with the acquisition of data

**Giorgia Querin**	Neurology Department, Pitié Salpêtrière-Charles Foix Hospital Group, AP-HP, Sorbonne University, Paris, France	Sub Investigator	Supported with the acquisition of data
**Konrad Rejdak**	Department of Neurology, Medical University of Lublin, Lublin, Poland	Principal Investigator	Supported with the acquisition of data
**Kourosh Rezania**	University of Chicago, Chicago, IL, USA	Principal Investigator	Supported with the acquisition of data
**Elena Rinaldi**	Fondazione Istituto di Ricovero e Cura a Carattere Scientifico, Istituto Neurologico Carlo Besta, Italy	Study Coordinator	Supported with the acquisition of data
**Rita Rinaldi**	IRCCS Istituto delle Scienze Neurologiche di Bologna, Bologna, Italy	Principal Investigator	Supported with the acquisition of data
**Michael H. Rivner**	Department of Neurology, Augusta University, Augusta, GA, USA	Principal Investigator	Supported with the acquisition of data
**Annekathrin Roediger**	Klinik für Neurologie, Universtitätsklinikum Jena, Jena, Germany	Sub Investigator	Supported with the acquisition of data
**Laura Rosow**	University of California, San Francisco Medical Center, San Francisco, CA, USA	Sub Investigator	Supported with the acquisition of data
**Simone Rossi**	IRCCS Istituto delle Scienze Neurologiche di Bologna, Bologna, Italy	Sub Investigator	Supported with the acquisition of data
**Elena Rossini**	AOU Sant'Andrea Roma, Italy	Sub Investigator	Supported with the acquisition of data
**Stephen Ryan**	University of Kentucky, Lexington, KY, USA	Sub Investigator	Supported with the acquisition of data
**Lotte Sahin Levison**	Aarhus Universitetshospital, Denmark	Sub Investigator	Supported with the acquisition of data
**Albert Saiz**	Servicio de Neurología ICN, Hospital Clinic de Barcelona, Universitat de Barcelona, Barcelona, Spain	Principal Investigator	Supported with the acquisition of data
**Maria Salvado**	Vall d'Hebron University Hospital, Passeig de la Vall d'Hebron, Barcelona, Spain	Sub Investigator	Supported with the acquisition of data
**Daniel Sánchez-Tejerina**	Hospital Universitari Vall D Hebron, Spain	Sub Investigator	Supported with the acquisition of data
**Margret Schwarz**	Klinik für Neurologie, Universitätsmedizin Göttingen, Göttingen, Germany	Study Coordinator	Supported with the acquisition of data
**María Sepúlveda**	Servicio de Neurología ICN, Hospital Clinic de Barcelona, Universitat de Barcelona, Barcelona, Spain	Sub Investigator	Supported with the acquisition of data

**Khema R. Sharma**	University of Miami Miller School of Medicine, Miami, FL, USA	Principal Investigator	Supported with the acquisition of data
**Sheetal Shroff**	Houston Methodist Hospital, Houston, TX, USA	Principal Investigator	Supported with the acquisition of data
**Olga Sidorova**	Vladimirsky Moscow Regional Research and Clinical Institute, Moscow, Russia	Sub Investigator	Supported with the acquisition of data
**Guilhem Solé**	Neuromuscular Reference Center AOC, Nerve-Muscle Unit, Bordeaux University Hospitals, Bordeaux, France	Principal Investigator	Supported with the acquisition of data
**Javier Sotoca**	Vall d'Hebron University Hospital, Passeig de la Vall d'Hebron, Barcelona, Spain	Sub Investigator	Supported with the acquisition of data
**Mads Stemmerik**	Copenhagen Neuromuscular Center, Department of Neurology, Rigshospitalet, University of Copenhagen, Copenhagen, Denmark	Sub Investigator	Supported with the acquisition of data
**Aleksandar Stojanov**	Clinic for Neurology, Clinical Centre Nis, Nis, Serbia	Sub Investigator	Supported with the acquisition of data
**Tanya Stojkovic**	Neurology Department, Pitié Salpêtrière-Charles Foix Hospital Group, AP-HP, Sorbonne University, Paris, France	Sub Investigator	Supported with the acquisition of data
**Kai Su**	University of Kentucky, Lexington, KY, USA	Study Coordinator	Supported with the acquisition of data
**Sebastian Szklener**	Department of Neurology, Medical University of Lublin, Lublin, Poland	Sub Investigator	Supported with the acquisition of data
**Alexander Tsiskaridze**	Pineo Medical Ecosystem, Tbilisi, Georgia	Principal Investigator	Supported with the acquisition of data
**Laura Tufano**	AOU Sant'Andrea Roma, Italy	Sub Investigator	Supported with the acquisition of data
**Michaela Tyblova**	Department of Neurology and Center of Clinical Neuroscience, First Faculty of Medicine Charles University and General Hospital, Prague, Czech Republic	Principal Investigator	Supported with the acquisition of data
**Eiko Uenaka**	Department of Neurology, Osaka University Hospital, Osaka, Japan	Study Coordinator	Supported with the acquisition of data
**Astrid Unterlauft**	Klinik und Poliklinik für Neurologie, Universitätsklinikum Leipzig A.ö.R, Leipzig, Germany	Sub Investigator	Supported with the acquisition of data
**Gabriel Valero**	Multiple Sclerosis and Clinical Neuroimmunology Unit, Neurology Department, Hospital Clínico Universitario Virgen de la Arrixaca, Murcia, Spain	Sub Investigator	Supported with the acquisition of data
**Fiammetta Vanoli**	Fondazione Istituto di Ricovero e Cura a Carattere Scientifico, Istituto Neurologico Carlo Besta, Italy	Sub Investigator	Supported with the acquisition of data

**Tamar Vashadze**	Pineo Medical Ecosystem, Tbilisi, Georgia	Sub Investigator	Supported with the acquisition of data
**Nuria Vidal Fernández**	Hospital de la Santa Creu i Sant Pau, Barcelona, Spain	Study Coordinator	Supported with the acquisition of data
**Marie-Hélène Violleau**	Neuromuscular Reference Center AOC, Nerve-Muscle Unit, Bordeaux University Hospitals, Bordeaux, France	Study Coordinator	Supported with the acquisition of data
**Nicolas Weiss**	Neurology Department, Pitié Salpêtrière-Charles Foix Hospital Group, AP-HP, Sorbonne University, Paris, France	Sub Investigator	Supported with the acquisition of data
**Nanna Witting**	Copenhagen Neuromuscular Center, Department of Neurology, Rigshospitalet, University of Copenhagen, Copenhagen, Denmark	Sub Investigator	Supported with the acquisition of data
**Jiann-Horng Yeh**	Department of Neurology, Shin Kong Wu Ho-Su Memorial Hospital, Taipei, Taiwan	Principal Investigator	Supported with the acquisition of data
**Leila Zaidi**	Centre de Référence des Maladies Neuromusculaires NEIdF, Département de Neurologie, Hôpital de Hautepierre, Centre Hospitalier Universitaire de Strasbourg and ERO-NMD Strasbourg, Strasbourg, France	Study Coordinator	Supported with the acquisition of data
**Leonid Zaslavskiy**	St. Petersburg Regional Clinical Hospital, Russia	Principal Investigator	Supported with the acquisition of data
**Jana Zschüntzsch**	Klinik für Neurologie, Universitätsmedizin Göttingen, Göttingen, Germany	Principal Investigator	Supported with the acquisition of data
